# Spotlight commentary: REBUS and the anarchic brain

**DOI:** 10.1093/nc/niaa007

**Published:** 2020-06-12

**Authors:** Tehseen Noorani, Ben Alderson-Day

**Affiliations:** n1 Department of Anthropology, Durham University, South Road, Durham DH1 3LE, UK; n2 Department of Psychology, Durham University, South Road, Durham, DH1 3LE, UK

**Keywords:** psychedelic, predictive processing, REBUS, context, default mode network, altered states

## Abstract

In ‘REBUS and the Anarchic Brain: Towards a Unified Model of the Brain Action of Psychedelics’, Carhart-Harris and Friston offer an important analysis of what the predictive processing framework has to offer our understanding of psychedelic experiences, providing an invaluable ground for psychedelic psychiatry. While applauding this, we encourage paying greater attention to contextual factors shaping extreme experiences and their sequalae, and suggest that the authors’ comparisons with certain non-psychedelic altered states may overlook more informative parallels that can be drawn elsewhere. Addressing both points will prove fruitful, ultimately, in identifying the mechanisms of action of greatest interest in psychedelic experiences.

In ‘REBUS and the Anarchic Brain: Towards a Unified Model of the Brain Action of Psychedelics’, Carhart-Harris and Friston (CH&F) present a model of relaxed beliefs under psychedelics (REBUS) and the anarchic brain. In relation to psychedelia, predictive coding approaches represent the latest shift in use of technological metaphors that have included Aldous Huxley’s reducing ‘valve’ and Timothy Leary’s eight ‘circuits’ of the brain. By analysing psychedelic experiences and analogous altered states in terms of predictive processing, CH&F integrate their respective research into the entropic brain hypothesis and the free-energy principle (317). We applaud this article as a seminal attempt to establish what the predictive processing framework (PPF) has to offer our understanding of the psychedelic experience, providing an invaluable ground for psychedelic psychiatry research moving forwards. At the same time, we caution that it may recapitulate extant biases in scientific research into experience. In particular, we are concerned that (i) insufficient attention is given to the practices and frameworks utilized when entering into extreme experiences, and (ii) overly simplistic comparisons with certain non-psychedelic altered states may obscure and overlook some more informative parallels that can be drawn elsewhere. We suggest that addressing both points will prove fruitful, ultimately, in identifying the mechanisms of action of greatest interest in the psychedelic experience.

Much of CH&F’s review centres on the role of different brain networks in the psychedelic state, as viewed through the lens of the free-energy principle. The ‘default mode network’ (DMN), central to this analysis, is typically associated with internally focused attention, and subjective states such as rumination and reflection. Psychedelics have been shown in recent years to disrupt the functioning of the DMN. Citing research suggesting the main hubs of the DMN are functionally positioned as far as possible from sensorimotor input (Margulies *et al.* 2016), the authors propose that the DMN corresponds to the highest levels of prior expectation and knowledge; accordingly, the authors take the DMN to be central to the seat of the self. This assumption shapes how psychedelic mechanisms of action are recast within the PPF:

…we propose that the brain’s highest levels provide an implicit, centralizing, and generalized compression of (potential) information held within and processed by subordinate levels; i.e., the high or deep hierarchical levels furnish beliefs about abstract, domain-general narratives regarding the world qualities *of self and states of being* (323, our emphasis).

However, the DMN has been implicated in a vast range of experiences and conditions, including almost every broad category of pathology. This has made it useful for constructing new hypotheses and theories (Alderson-Day and Callard 2016) – but mysterious objects of investigation can be all things to all researchers. For instance, the DMN has been closely associated with how we think about others, supporting theory of mind, the narrative self and self-other judgements central to social functioning (Spreng and Andrews-Hanna 2015). Rather than looking to the self or ego, we wonder whether the REBUS model may be just as enriched by *social* identity, personhood or belonging at the top of a proposed ‘hierarchy’ of priors (Moutoussis *et al.* 2014). Put another way, are we the driver of our core expectations, or are others?

One of the appeals of the PPF is its ability to account for subjective experience beyond the confines of the individual. Given the call to move beyond individualistic research paradigms from prominent researchers within psychedelic science (e.g. Vollenweider 2017), it is a lost opportunity for the authors to imply that hierarchical models of the mind end with the individual. Instead, we might look for how the conception of self is shaped at every level by one’s social interactions and ideas of a ‘social other’.

Attending to contextual factors is a well-trodden path in relation to psychedelic drug action. Over 35 years ago, Dobkin de Rios (1984, 4) proposed ‘to build a theory of drug effects that takes into account the importance of antecedent cultural variables so as to predict such effects’. Today it is accepted that context is central to the psychedelic experience, and the bulk of psychedelic researchers and therapists consider the therapeutic scaffolding of the psychedelic experience to be central in constituting the experience.

Yet it remains difficult to gain objective data about psychedelic effects in contexts outside of tightly regulated clinical administration (*cf*. Studerus *et al.* 2012). In their reconceptualization of the psychopharmacological doxa of ‘set and setting’ through the PPF, CH&F note that psychedelic effects result from more than the mere loosening of priors (e.g. they can come from the limbic system or interoceptive signals). However, we suggest they do not pay enough attention to the expectation-constituting role that contextual factors play in shaping priors, for not only surprising or insightful, but also *confirmatory* experiences. Given how important preparation, set and setting are for the impressive clinical outcomes, and the centrality of integration in the emerging consensus amongst psychedelic researchers that ‘there is no such thing as a bad trip’, it is a strange omission that the authors do not attend more to normative and social drivers when theorizing how people respond to psychedelics.

Psychedelic researchers could learn from hallucinations researchers’ interest in developing models beyond the confines of the acute experiences, such as ‘non-clinical’ voice-hearers. For example, Luhrmann (2012) has documented how evangelicals learn to hear the voice of God, precisely because of a pre-existing framework that identifies any voices experienced as not coming from the self as coming instead from God (on non-clinical voice-hearing, see also Powers *et al.* 2017). This may also take deliberate and repeated practice – building a strong set of socially situated expectations about the oncoming wave of the ‘unexpected’. Analogies can be drawn to the frameworks delimiting the effects of drug administration, which are conferred by the preparation and context of psychedelic drug use. Luhrmann’s analysis illustrates the value of understanding pre-existing frameworks delimiting the set of expected possibilities (and impossibilities) before one enters into an altered state.

By not giving context its due, CH&F risk drawing some parochial conclusions, such as that psychedelic experiences bring about the loosening of ‘partisan and/or overly-confident political, religious and/or philosophical perspectives’ (317). This is a commonly held view, but it is contradicted by historical and cross-cultural evidence of the use of psychedelics in *shoring up* strong belief systems (e.g. Piper 2015). Taking leave from the structural analogy of intention and preparation in evangelicals, could the effects of psychedelics be equally well understood as the strengthening of healthy and intention-laden priors as the loosening of pathogenic ones?

Instead of comparing the psychedelic experience with those groups who have voluntarily chosen to entered altered and unusual states, CH&F’s primary point of comparison is with the early stages of psychosis. In contrast to recent approaches which posit a role for strong priors in the development of psychosis (Adams *et al*. 2013, see also in relation to hallucinations, Corlett *et al.* 2018), they propose that psychedelic states and early psychotic experiences share a similar loosening of higher-level priors (with expectations becoming calcified only later for people with psychosis). Their reasoning for this lies in disruptions to a sense of self, and a generalized feeling of anxiety – an account which is reminiscent of the ‘basic symptoms’ of schizophrenia proposed by phenomenological psychopathology. We worry, though, that it again leaves out the role of the other, i.e. the social context, and emerging social threat, which features again and again in early psychosis. Here, the spiral of strong, negative expectations seems to provide more dis-analogy than analogy with transformative and positive psychedelic states.

The dis-analogy between psychedelic trial participants at the pre-dosing stage and people unaware of an upcoming psychotic break is important not just for the acute experience, but also for what comes afterwards. CH&F propose that any delusions that do occur in relation to psychedelics use only do so during the acute psychedelic experiences, as a way to ‘close out uncertainty’ (328) created by the relaxed precision weighting of priors. Yet the role of integration in the post-acute period following psychedelic administration is considered crucial for therapeutic gain, while the potential harms from no or poor-quality support is understudied, owing to a focus in research trials on isolating the effects of the drugs and ethical imperatives to provide substantial care in integrating psychedelic experiences. It is not known currently what role downstream support – either during integration practices or between successive psychedelic experiences – plays in the potential emergence of delusional ideation. Investigating this and its relationship with the naturalistic onset of delusions (e.g. Freeman *et al.* 2019) requires longitudinal research that carefully attends to the context of lived experience.

Psychedelic researchers will continue to wrestle with the tension between offering a parsimonious analytics to explain psychedelic experiences, and the diversity and elusiveness of the latter. Accordingly, we admire the scale of what CH&F offer in their REBUS model, while suggesting a need to rebalance – perhaps away from parsimony. In order to realize its potential to explain a range of unusual and fantastic states within a single framework, the PPF must take the social situatedness of experience seriously. This requires attending to the context of psychedelics use and integration in order to better understand whether and how expectations might be reinforced rather than confounded. We have suggested that comparison with non-clinical groups who seek out unusual states may be more fruitful in these regards, whereas a more detailed consideration of pathological states such as psychosis is liable to reveal dis-analogies. If the PPF does not grapple with such considerations, we risk conceptualizing the variety of psychedelic states – from visions to desires and musings to rapture – through a ‘desert landscape’ (Clark 2013, 200) of an individual’s prior and precision only. By contrast, any explanatory framework purporting to explain psychedelic experiences ought to retain their richness and transformative power. To this end, PPF advocates would do well to supply context for relaxed priors in terms of their content, particularly with regards to prior beliefs about the self in relation to others.
